# P-155. Challenges with Artemether and Lumefantrine in the Management of Uncomplicated Malaria Infection

**DOI:** 10.1093/ofid/ofae631.360

**Published:** 2025-01-29

**Authors:** Flora Olcott

**Affiliations:** University College London - Institute of Global Health, London, England, United Kingdom

## Abstract

**Background:**

Annually there are approximately 1,500 cases of imported malaria in the UK, with the majority being falciparum infection in African diaspora visiting friends and relatives (VFR). The management of uncomplicated falciparum infection usually entails a treatment course of artemether and lumefantrine. However, this medication can pose challenges to prescribers and patients alike due to the dosing schedule and administration requirements.

We assessed the management of uncomplicated falciparum malarial infection in the emergency department and designed a tailored treatment package for patients being managed in an outpatient setting in order to improve medication compliance and clinical outcome for this cohort.

Compliance Aid for Artemether/Lumafantrine - Patient Information Leaflet

**Figure:**
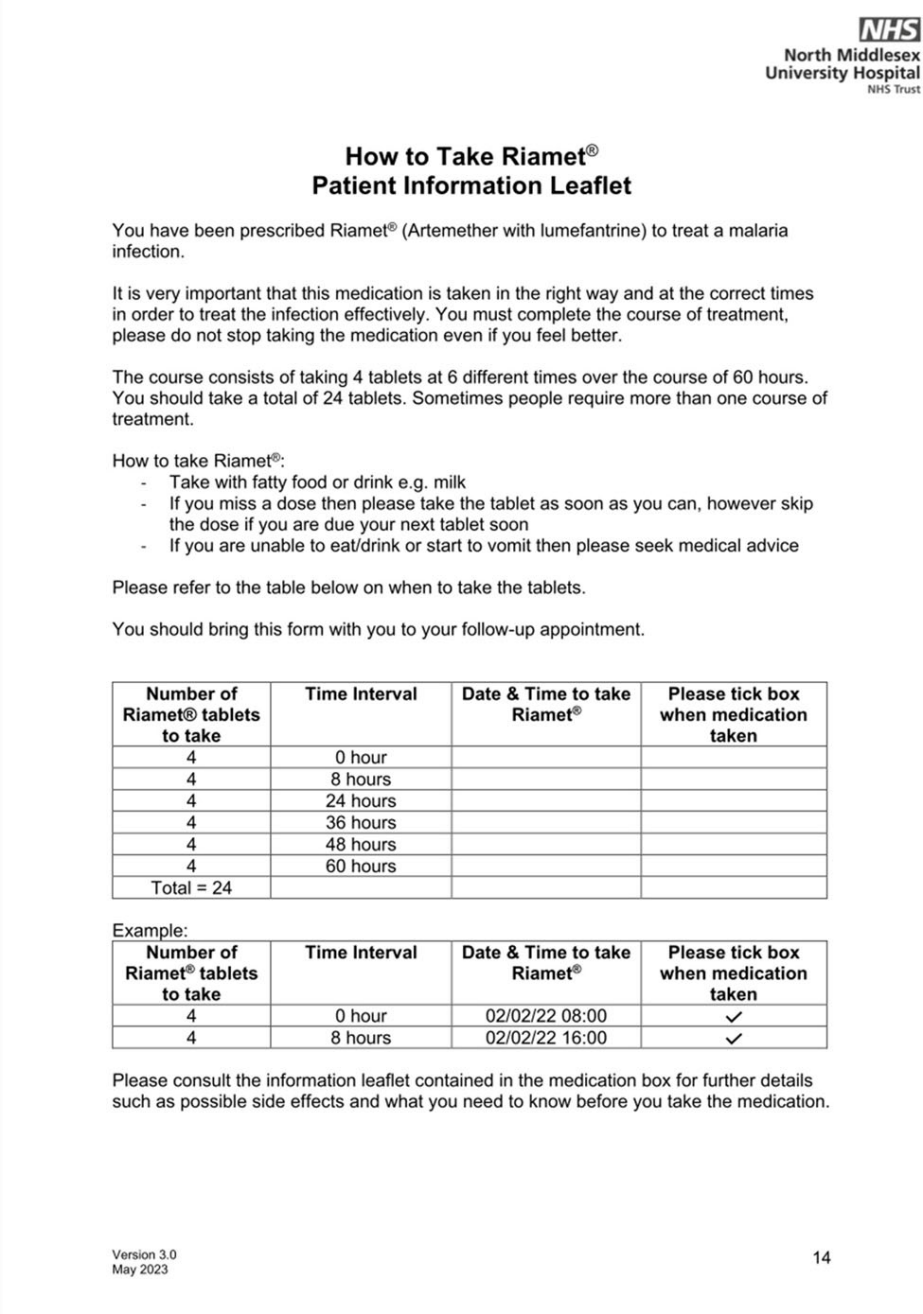

Customised patient information leaflet to be completed by prescriber and given to patient to improve medication adherence

**Methods:**

A retrospective audit was conducted at a hospital in London from December 2021 to January 2023. The management of uncomplicated falciparum malarial infections and the adequacy of existing clinical pathways were assessed using electronic patient records.. Patients that presented to the emergency department, diagnosed with uncomplicated falciparum infection and discharged home with oral treatment with subsequent outpatient clinic follow-up were included

**Results:**

A total of 17 patients were included in the audit. 41% (7/17) patients were identified as experiencing issues with medication including confusion regarding when to take tablets, not taking medication with food and not completing the course due to symptom resolution. 3 patients consequently required further course of Riamet and 1 person required admission to hospital due to rising parasitaemia as a result of not taking artemether and lumefantrine correctly. As a result of these findings, a customised patient information leaflet and artemether and lumefantrine prepacks were created and made available in the emergency room.

**Conclusion:**

This study demonstrates the challenges posed by artemether and lumefantrine for prescribers and patients. Patients with falciparum infection are at risk of rapid deterioration and therefore clear instructions on when and how to take the medication are needed. Follow-up is essential in this cohort and medication compliance issues should be considered in persistent/rising parasitaemia or ongoing symptoms.

**Disclosures:**

**All Authors**: No reported disclosures

